# Combining Citizen Science and Genomics to Investigate Tick, Pathogen, and Commensal Microbiome at Single-Tick Resolution

**DOI:** 10.3389/fgene.2019.01322

**Published:** 2020-01-21

**Authors:** Gaurav Chauhan, Jesse McClure, Jessica Hekman, Patrick W. Marsh, Jeffrey A. Bailey, Rachel F. Daniels, Diane P. Genereux, Elinor K. Karlsson

**Affiliations:** ^1^ Bioinformatics and Integrative Biology Program, University of Massachusetts Medical School, Worcester, MA, United States; ^2^ Vertebrate Genomics, Broad Institute of MIT and Harvard, Cambridge, MA, United States; ^3^ Department of Pathology and Laboratory Medicine, Warren Alpert Medical School, Brown University, Providence, RI, United States; ^4^ Department of Immunology and Infectious Diseases, Harvard T.H. Chan School of Public Health, Boston, MA, United States; ^5^ Program in Molecular Medicine, University of Massachusetts Medical School, Worcester, MA, United States

**Keywords:** tickborne disease, genomics, microbiome, disease transmission, host-pathogen, citizen science, lyme disease, tick vector

## Abstract

The prevalence of tickborne diseases worldwide is increasing virtually unchecked due to the lack of effective control strategies. The transmission dynamics of tickborne pathogens are influenced by the tick microbiome, tick co-infection with other pathogens, and environmental features. Understanding this complex system could lead to new strategies for pathogen control, but will require large-scale, high-resolution data. Here, we introduce Project Acari, a citizen science-based project to assay, at single-tick resolution, species, pathogen infection status, microbiome profile, and environmental conditions of tens of thousands of ticks collected from numerous sites across the United States. In the first phase of the project, we collected more than 2,400 ticks wild-caught by citizen scientists and developed high-throughput methods to process and sequence them individually. Applying these methods to 192 *Ixodes scapularis* ticks collected in a region with a high incidence of Lyme disease, we found that 62% were colonized by *Borrelia burgdorferi*, the Lyme disease pathogen. In contrast to previous reports, we did not find an association between the microbiome diversity of a tick and its probability of carrying *B. burgdorferi*. However, we did find undescribed associations between *B. burgdorferi* carriage and the presence of specific microbial taxa within individual ticks. Our findings underscore the power of coupling citizen science with high-throughput processing to reveal pathogen dynamics. Our approach can be extended for massively parallel screening of individual ticks, offering a powerful tool to elucidate the ecology of tickborne disease and to guide pathogen-control initiatives.

## Introduction

Tickborne diseases (TBDs) are an emerging public health threat in the United States and worldwide (Institute of Medicine, 2011; Paules et al., 2018) (**Table 1**). The US Centers for Disease Control and Prevention (CDC) lists seven tick species known to transmit 19 human pathogens in the US alone (Centers for Disease Control and Prevention, 2019). The CDC recorded a total of 59,349 confirmed and probable TBD cases in 2017, up from 22,527 cases in 2004. The true burden of tickborne infections is estimated to exceed reported cases by as much as 10-fold (Nelson et al., 2015; Schiffman et al., 2018).

**Table 1:** CDC reports of tickborne disease caused by bacterial and protozoan pathogens increased markedly from 2004 to 2016 (Rosenberg et al., 2018).

Disease	Year		% increase	Pathogens	Tick vectors
	2004	2016			
Lyme disease	19,804	36,429	84%	*Borrelia burgdorferi, B. mayonii*	Western blacklegged tick (*Ixodes pacificus*), Blacklegged tick (*Ixodes scapularis*)
Tularemia	134	230	72%	*Francisella tularensis*	American dog tick (*Dermacentor variabilis*), Wood tick (*Dermacentor andersoni*), Lone star tick (*Amblyomma americanum*)
Spotted fever rickettsiosis	1713	4269	149%	*Rickettsia rickettsii. Rickettsia parkeri, Rickettsia sp 364D; Rickettsia akari*	American dog tick (*Dermacentor variabilis*), Wood tick (*Dermacentor andersoni*), Brown dog tick (*Rhipicephalus sanguineus*)
Anaplasmosis/Ehrlichiosis	875	5750	557%	*Anaplasma phagocytophilum, Ehrlichia chaffeensis, E. ewingii, E. muris eauclairensis*	Western blacklegged tick (Ixodes pacificus), Blacklegged tick (Ixodes scapularis), Lone star tick (Amblyomma americanum)
Babesiosis**	1126	1910	70%	*Babesia microti, B. divergens, B. duncani*, strain MO-1	Blacklegged tick (*Ixodes scapularis*)

**Cases reported in 2011. No data for 2004.

In parallel, the geographic distribution of ticks and the pathogens they carry is expanding, and new pathogens continue to emerge (Paddock et al., 2016; Rosenberg et al., 2018). *Ixodes scapularis*, the blacklegged tick that transmits Lyme Disease in the Eastern United States, is now considered established in twice as many U.S. counties as in 1998, and Lyme disease is now widespread throughout New England and the Midwest (Eisen et al., 2016). Moreover, cases of spotted fever rickettsiosis (SFR) have been reported throughout the contiguous United States. In addition, eight new tickborne pathogens (TBPs) have been identified since 2010 in the US (Vayssier-Taussat et al., 2013; Bonnet et al., 2014; Tokarz et al., 2014). Indeed, it is estimated that up to half of all TBDs may be caused by pathogens not previously known to be associated with ticks, or not yet identified at all (Feder et al., 2007).

Given the substantial and increasing impact of TBDs on human health and expanding geographic ranges of ticks and the associated pathogens, there is an urgent need for effective control strategies. Unfortunately, diverse strategies including public education campaigns to raise awareness of tick disease (Beaujean et al., 2016), targeted culling of mammalian species that help sustain tick populations (Jordan et al., 2007; Telford, 2017), vaccination against TBDs (Beaujean et al., 2016; Richardson et al., 2019), and application of pesticides have failed to produce substantial, durable declines in the incidence of TBD, and have sometimes faced public skepticism (Najjar et al., 2017).

Approaches that leverage detailed information about the vector microbiome have shown promise for control of pathogens transmitted by other arthropods. For example, modulating the mosquito microbiome reduces transmission of Malaria, Dengue, and other diseases (Durvasula et al., 1997; Moreira et al., 2009; Mousson et al., 2010; Hughes et al., 2011; Walker et al., 2011; Dutra et al., 2016). Like other arthropods, ticks are inhabited by communities of commensal and symbiotic microorganisms that influence, or even drive, pathogen transmission (Clay et al., 2008; Clay and Fuqua, 2010; Narasimhan et al., 2014; Bonnet et al., 2017), suggesting that microbiome-based strategies could also be useful in addressing TBDs.

Developing microbiome-focused approaches requires understanding how the vector microbiome modulates the probability of pathogen carriage and transmission. The tick microbiome has been found to vary with tick sex, species, and geographic location (Van Treuren et al., 2015), but many studies have pooled ticks before processing, precluding insight into individual-level dynamics of tick-pathogen associations (Vayssier-Taussat et al., 2013; Salkeld et al., 2014; Tokarz et al., 2014). Large-scale, multistate tick studies, including some using citizen science, have examined infection with pathogens, but did not profile the commensal microbiome (Stromdahl et al., 2001; Diuk-Wasser et al., 2012; Lee et al., 2014; Sayler et al., 2017; Nieto et al., 2018). Studies that included microbiome profiling have generally focused on relatively small numbers of ticks collected in limited geographic areas. These studies provide critical insight into tick-associated viruses (Tokarz et al., 2014), vertical transmission (Clay et al., 2008), and host-pathogen concordance (Rynkiewicz et al., 2015), but precluding inference of broader patterns.

Here, we describe Project Acari, a program designed to collect microbiome and pathogen data from large numbers of individual ticks collected from both broad geographic areas and over extended time periods. Citizen scientists mail unattached ticks they find during their normal activities, each labeled by geographic location, using our inexpensive “Tick Kit”. We then extract and process DNA in 96-well plates, attaching sequence barcodes that uniquely identify the tick, microbiome, and pathogen DNA extracted from each tick. This allows us to analyze the co-occurrence of pathogens and particular microbiome species with single-tick resolution. Applying this approach to a pilot study of *I. scapularis* ticks collected in Grafton, Massachusetts revealed a previously unknown association between pathogen infection and particular components of the microbiome, demonstrating the potential of this approach to enable discoveries when scaled up to tens of thousands of ticks collected from a broad geographic area over extended periods of time.

## Materials and Methods

### Sample Collection

We used two different strategies for tick collection. First, for our pilot study of *I. scapularis* ticks, we needed a sample set with high proportion of pathogen-infected *I. scapularis* ticks. We asked a small cohort of volunteers living in an area of Massachusetts with high reported incidence of Lyme disease to place ticks they found, if unattached, in a resealable zipper storage bag. These ticks were delivered directly to our research lab by the participants in November 2017. We confirmed by visual inspection that all collected ticks were adult *I. scapularis*.

**Figure 1:**
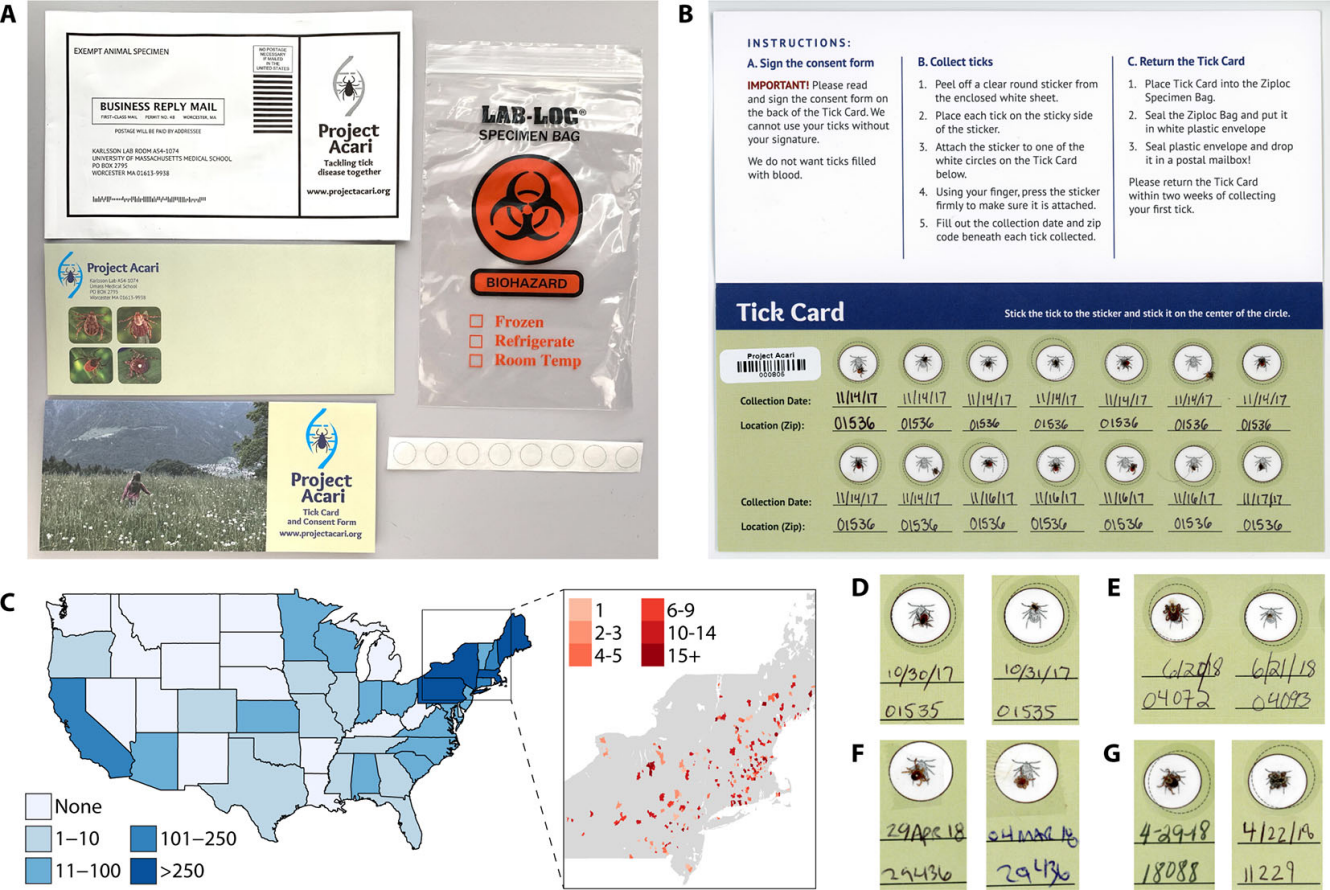
Citizen science approach enables large-scale collection of many tick species from a broad geographic area. **(A)** Our custom-designed “Tick Kit” includes a Tick Card, clear stickers to adhere ticks to the Tick Card, as well as a sealable biohazard bag and prepaid plastic envelope for return shipping. All components fit into standard first-class mail envelopes for fast and inexpensive shipping in both directions. **(B)** A returned Tick Card with 14 ticks, the maximum capacity of the Tick Card, each sealed on the card with a clear sticker and labelled by the volunteer with collection data and location (zip code). Each Tick Card has a unique barcode and instructions are printed on the card adjacent to the tick collection section. **(C)** Over 900 people volunteered for Project Acari during two recruitment windows (October and November of both 2017 and 2018); we received 322 Tick Cards with 2,417 ticks from 32 states. Most (2,027 ticks) came from states in the northeast (inset), the geographic region with the highest rates of TBD in the United States. **(D)** Visual inspection of a subset of tick cards suggested that, while most of the ticks collected were adult female (left) or male (right) *Ixodes scapularis*, we also received **(E)** diverse life stages, including adults (left) and nymphs (right) as well as other species, including **(F)** female and male *Amblyomma americanum* (lone star tick) and **(G)** female and male *Dermacentor variabilis* (American dog tick).

Second, to expand the geographic range of the project, we developed a “Tick Kit” that was inexpensive to produce and ship and that conformed to the dimensions and requirements for standard U.S. Postal Service First Class Mail Letters. Each Tick Kit costs $0.60 and includes 1) a Tick Card with individual spots for 14 ticks; 2) stickers to affix and seal the ticks to the Tick Card; 3) a sealable biohazard-labeled polypropylene bag to hold the Tick Card after tick collection; and 4) a pre-paid postage sealable return envelope to send the Tick Card to us (**Figure 1A**). Each Tick Card is uniquely barcoded for easy tracking.

To maximize the collection of adult ticks, which have the greatest probability of carrying at least one species of pathogen and are easiest to see and are, therefore, the easiest to collect, we emphasized the collection of ticks in the late fall. Project Acari started enrolling participants in October 2017 and sent kits out in October and November of both 2017 and 2018. We advertised the project through social media (Facebook, Instagram, and Twitter), and through email to the community of citizen scientists enrolled in the Darwin's Ark project (DarwinsArk.org). People interested in participating visited our website (ProjectAcari.org) to register and request a Tick Kit. Participation is free.

Upon receipt from citizen scientists, each Tick Kit is incubated at -20°C for 24 h to ensure that no ticks are alive. Each Tick Card is then scanned, its image saved, and stored at -20°C until processing. We store information on each tick, including collection location (as zip code), date of collection, and date of receipt, in a custom SQL database. We update the database to indicate tick species, infection status, and microbiome profile as information becomes available.

### High-Throughput DNA Extraction

We developed a method for efficient extraction of DNA from large numbers of individual ticks. Initially, we tried bead beating, the standard approach for extracting tick DNA. While this method is effective for extraction from small numbers of ticks in screw-cap tubes using homogenization speeds ~4000 rpm, it could not be scaled up to permit extraction within the 96-well format, and so was not amenable for higher-throughput processing. Commercially available homogenizers that accomodate 96-well plates generally have a maximum speed of 1,600 rpm. At these speeds, the hard exoskeletons of ticks remain intact after 10 min of bead beating.

We therefore developed an alternative approach to address this challenge. We first froze the ticks by placing the 96-well plate containing the ticks in liquid nitrogen, then manually homogenized the ticks using the 96-well Zymo Research Squisher-96. Individual ticks were placed into each well of a 96-well deep-well plate (Squisher-96, Zymo research Cat No.: H1004), then washed with 70% ethanol followed by three washes with sterile water. Next, the Squisher-96 tool was inserted into plates, which were then placed in a container filled with liquid nitrogen and incubated for 5–10 min. The frozen ticks were completely crushed and homogenized using the Squisher-96 tool and incubated in Buffer ATL and proteinase K (Qiagen Blood and Tissue Kit, Cat No.: 69581) for 3 h at 55°C according to the manufacturer's instructions. The DNA was then extracted following the manufacturer's protocol. The DNA concentration was measured using a NanoDrop™ spectrophotometer (Thermo Fisher Scientific, Waltham, MA USA). To avoid cross-contamination, each Squisher-96 was used only once and care was taken to avoid splash and aerosolization during homogenization.

### Analysis of *Ixodes Scapularis* Ticks

#### Screening for B. burgdorferi

We screened for *B. burgdorferi* senso lato by polymerase chain reaction (PCR), using primers specific to Outer Surface Protein A (ospA2 and ospA4) and Flagellin (fla1 and fla3) (Persing et al., 1990). Each 20 μl PCR reaction mixture contained 25 ng genomic DNA, 200 μM of each dNTP, 0.5 μM each forward and reverse primers, 1 μl HotStarTaq DNA Polymerase (Qiagen Cat No.: 203203), and 1X PCR buffer. Thermal amplification was performed in an Eppendorf Mastercycler Pro S (Eppendorf North America, Inc.) using the following cycling conditions: 15 min of initial denaturation at 94°C followed by 25 cycles consisting of denaturation at 94°C for 0.5 min, annealing at 55°C for 0.5 min, and extension at 72°C for 1 min, with a final extension at 72°C for 10 min and indefinite hold at 4°C. The resulting amplicons were visualized using SYBR Gold on a 2% agarose gel following electrophoresis.

#### 16S rDNA library preparation

To sequence the microbiome of each tick, we amplified the V3–V4 region of 16S rDNA and added barcodes and Illumina adapters according to Illumina's protocol for 16S Metagenomic Sequencing Library Preparation (Illumina, 2013). To minimize carry-over contamination, separate rooms were designated for handling of pre- and post-PCR samples. The resulting libraries were quantified using a Quant-iT™ PicoGreen™ dsDNA Assay Kit (ThermoFisher Scientific Cat No.: P11496). For the 128 libraries (99 females, 29 males; 88 infected, 40 uninfected) yielding DNA concentrations above 10 nM, we normalized the concentrations to 4 nM and pooled the samples in equimolar concentrations. We sequenced the amplicons using an Illumina Miseq v3 reagent kit and 10% PhiX, following the standard protocol for 16S sequencing. The resulting sequence reads were filtered for read quality, base-called, and demultiplexed using bcl2fastq (v2.20).

#### Quality control and filtering of 16S sequencing reads

We imported and processed the demultiplexed paired-end sequencing reads using the ‘quantitative insights into microbial ecology' pipeline of Qiime2 v.2018.2.0 (Bolyen et al., 2019). The reads were denoised using the “divisive amplicon denoising algorithm” DADA2 (Callahan et al., 2016) plugin in Qiime2. This step filtered out noise and corrected errors in marginal sequences, removed chimeric sequences and singletons, merged paired-end reads, and finally dereplicated the resulting sequences, resulting in high-resolution amplicon sequence variants (ASVs) for downstream analysis. ASVs assume that biological sequences are present in the sample and can resolve single-nucleotide differences in sequence variants. Using the “denoise-paired” command, the DADA2 options passed were trim-left-f: 17, trim-left-r: 21, trunc_len_f: 300, trunc_len_r: 240, with all other options left as default. Additionally, ASVs with a minimum sample frequency of 2 and a total minimum frequency of 10 were removed.

#### Assessment of overall bacterial diversity

We assessed bacterial diversity within [alpha diversity: observed ASVs, Shannon index (Shannon, 1948) and Faith PD index (Faith, 1992)] and between (beta diversity: weighted (Lozupone et al., 2007) and unweighted UniFrac (Lozupone and Knight, 2005) ticks using the q2-diversity plugin in QIIME2. Principal coordinates plots of beta diversity were visualized using the EMPeror tool (Vázquez-Baeza et al., 2013). PERmutational Multivariate ANalysis Of VAriance (PERMANOVA) analysis was used to measure the effect size and significance on beta diversity for grouping variables (Balakrishnan et al., 2014). As alpha and beta diversity metrics are sensitive to uneven sampling depths, multiple rarefactions were performed prior to computing the diversity indices. The number of ASVs per sample was randomly selected without replacement at an even depth of 0 to 20,000 sequences (**Figure S1**). For alpha and beta diversity analysis, the ASVs was rarefied at 8,333 sequences per sample.

#### Taxonomic annotation and relative abundance of identified bacterial taxa

We performed taxonomic annotation of ASVs in QIIME 2 using a pre-trained Naïve Bayes classifier and the q2-feature-classifier plugin (Bokulich et al., 2018). Prior to annotation, the classifier was trained on the QIIME-compatible 16S SILVA reference (99% identity) database v.128 (Quast et al., 2013). The reference sequences were trimmed to span the amplified v3-v4 region of the 16S rDNA gene using the extract-reads command of the q2-feature-classifier plugin in QIIME2. The resulting relative abundance tables of annotated ASVs were exported into (R Core Team, 2018) to generate stacked bar plots to visualize the relative abundance of bacterial taxa across sample types.

#### Identification of bacterial markers potentially associated with B. burgdorferi infection

To identify possible bacterial taxa associated with *B. burgdorferi* infection, we used the linear discriminant analysis (LDA) effect size method (LEfSe) v.1.7 (Segata et al., 2011). The relative abundance tables for the annotated ASVs were imported into LEfSe. *B. burgdorferi* infection was entered as the class vector (the main condition under investigation) and the sex of each tick as a subclass (as a biologically meaningful grouping within each class).

To identify associated taxa, LEfSe first uses pairwise non-parametric factorial Kruskal-Wallis sum-rank tests to detect ASVs with significant differential abundance between classes. Then, for biological consistency, it performs pairwise unpaired Wilcoxon rank-sum tests to compare differentially abundant ASVs from the previous step between subclasses (gender). The default p-value of <0.05 was used for both tests and ASVs with consistent significant differential abundance among the two classes and across both subclasses were considered possible taxa associated with *Borrelia* infection. Lastly, the associated taxa were used to build a latent Dirichlet allocation (LDA) model from which the relative differences among classes were ranked to obtain the effect size. The LDA model was built using default parameters and the resulting logarithmic scores of the analyses are presented. An LDA score of ≥2.0 was set as the threshold to retain taxa.

## Results

In the first phase of Project Acari, we sought to demonstrate the potential of large-scale, citizen science as a tool for investigating the dynamics of TBD. Our initial tick collection, focused on an area of Massachusetts with a high rate of Lyme disease, yielded the 192 *I. scapularis* ticks used in our pilot genomics project. We then extended Project Acari to the entire United States using mail-in Tick Kit cards provided for free. Finally, we developed high-throughput methods for processing ticks using 96-well plates, enabling rapid scale-up.

### Tick Collection by Citizen Scientists

We completed a two-stage trial launch in October 2017 and 2018, receiving requests for 1,027 kits. Our kit is designed to be inexpensive to produce and mail, and easy for citizen scientists to use and return (**Figure 1A**). Each kit has space for 14 ticks labeled with collection data and zip code. We specifically request unengorged ticks (**Figure 1B**). As of January 15, 2019, 31% (322) of kits have been returned from 302 zip codes and 32 states, with an average of 7.5 ticks/card. (**Figure 1C**). Of the 2,417 total ticks received, 2,171 (90%) were unengorged and 246 (9.9%) were engorged. Most (1996 ticks; 82.5%) were collected in the late fall season (August–November), and the remainder during the winter (December–March; 126 ticks) or spring (April–June; 295 ticks). Visual inspection of a subset of Tick Cards showed that while the majority of ticks appeared to be adult *I. scapularis*, the cards also included ticks of other life stages and other species, including *Dermacentor variabilis* (American dog tick), *Amblyomma americanum* (lone star tick), *Ixodes pacificus* (Western blacklegged tick), and *Rhipicephalus sanguineus* (Brown dog tick) (**Figure 1D–G**). In 29 zipcodes, ticks were collected in both 2017 and 2018, highlighting the potential for longitudinal sampling.

### Higher-Throughput Tick DNA Extraction

Our high throughput method enabled DNA extraction both from ticks collected locally through our pilot project and from ticks mailed on Tick Cards from various locations throughout the United States and then stored at -20°C for up to one year. Among the 192 adult *I. scapularis* ticks included in our pilot study, the DNA yield per tick ranged from 0.074 to 3 μg, averaging 1.033 from female ticks and 0.535 μg from the smaller male ticks. Among the 188 ticks received on Tick Cards mailed from across the United States, the yields ranged from 0.079 to 7.6 ug per tick, averaging 0.692 and 0.362 μg from female and male *Ixodes*, respectively. The average DNA yield from non-*Ixodes* ticks, which are generally larger, was 2.13 μg. We also successfully extracted RNA from Tick Card ticks, indicating that ticks collected by citizen scientists could also be used to survey for RNA viruses.

### Microbiome Sequencing Pilot Study

We used PCR to screen for *B. burgdorferi* in all 192 *Ixodes scapularis* ticks in our pilot study. We identified *B. burgdorferi* infection in 119 (61.9%) of them, consistent with infection rates reported in this region (Laboratory of Medical Zoology, U Mass Amherst, 2013). We prepared a 16S metagenomic sequencing library for each tick, then sequenced the 128 libraries with concentrations > = 10nM (99 females and 29 males; 88 positive for *B. burgorferi*, 40 negative). For each sample, we obtained an average of 32,629 DADA2 (Callahan et al., 2016)‐processed quality sequences (± 41,579 SD) and 66 (± 36 SD) unique ASVs. The rarefaction curve indicated sufficient sequencing depth to adequately capture the full diversity of ASVs in all samples sequenced (**Figure S1**).

#### Coinfection

We used the 16S sequencing data to identify ticks coinfected with multiple pathogens in addition to *B. burgdorferi*, in which infection was defined as at least 0.1% of microbiome reads coming from the TBP. Consistent with previous reports, we observed several cases of double and even triple infection (Hersh et al., 2014; Moniuszko et al., 2014; Prusinski et al., 2014). Six ticks were coinfected with *B. burgdorferi* and *B. miyamotoi*. Seven ticks were coinfected with *B. burgdorferi* and *Anaplasma phagocytophilum*, which can cause granulocytic anaplasmosis in humans, although our data does not distinguish the infectious and non-infection strains (Massung et al., 2002). Of these, two ticks carried all three of these pathogens. Because our PCR process targeted the eubacterial 16S region, our data do not indicate whether these ticks were also coinfected with *Babesia*, a eukaryotic pathogen previously reported to be present in coinfections (Krause et al., 1996; Swanson et al., 2006; Hersh et al., 2014).

#### Alpha diversity

**Figure 2:**
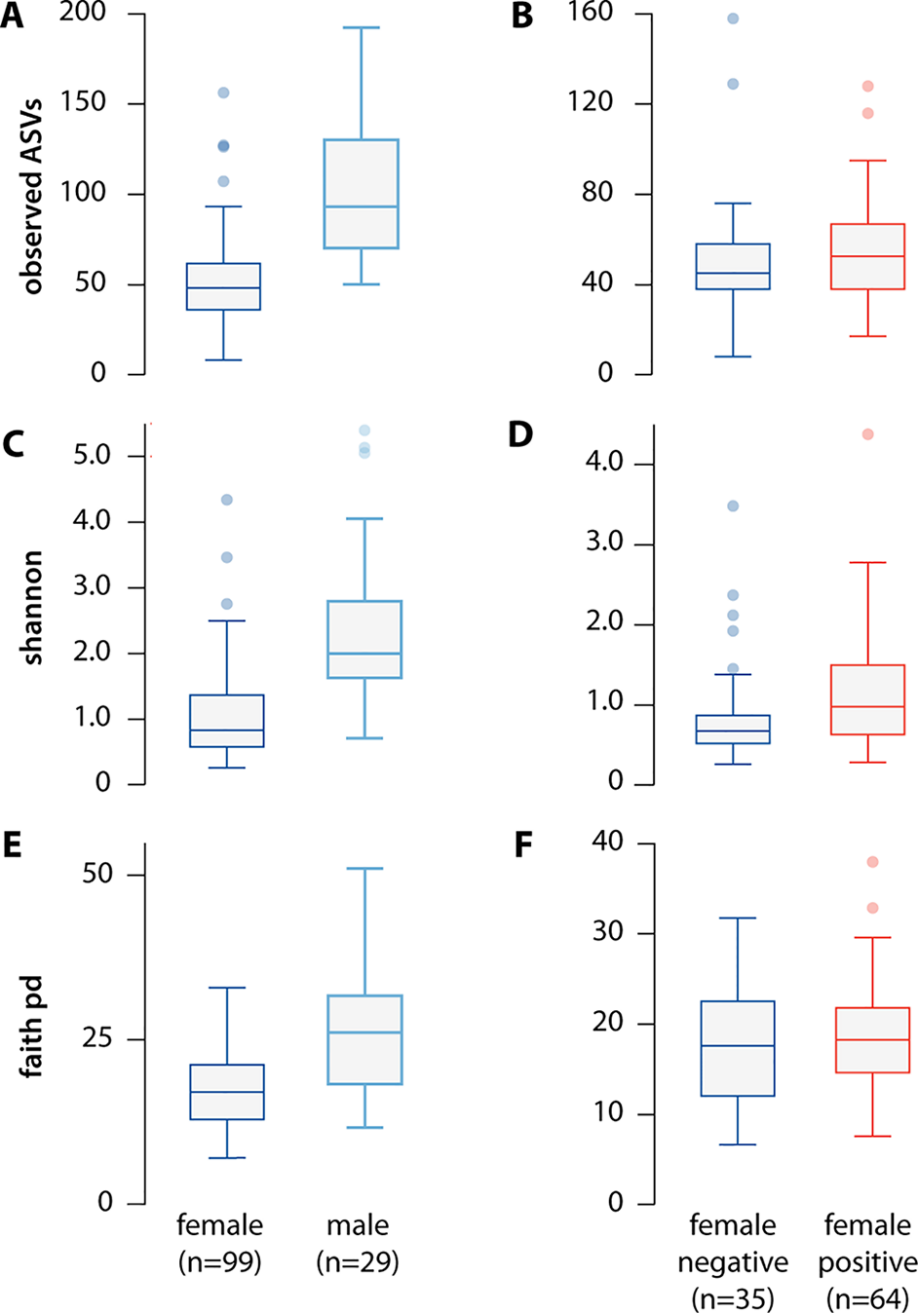
Alpha diversity of the microbiome differs significantly between females and males, but not between *B. burgdorferi-*infected and uninfected ticks. We measured alpha diversity using three different metrics, including **(A)** the count of observed ASVs by sex (p = 1.01e-10) and **(B)** by infection status (p = 0.30), **(C)** the Shannon index, a non-phylogenetic alpha diversity metric, by sex (p = 6.45e-10), and by **(D)** infection status (p = 0.02), and **(E)** Faith's Phylogentic Diversity index, a phylogenetic alpha diversity metric by sex (p = 6.45e-10) and **(F)** by infection status(p = 0.56). While infection status was significant using the Shannon Index, this difference disappeared after removing *B. burgdorferi* reads from the analysis (p = 0.58).

Consistent with previous reports, individual male ticks had greater microbial diversity than that in female ticks (Van Treuren et al., 2015; Thapa et al., 2018). Male ticks had a larger total number of microbial species (**Figure 2A**; p = 1.01e-10); higher Faith phylogenetic diversity index scores (Faith, 1992) (**Figure 2C**; p = 7.0e-6), a measure of total phylogenetic branch length spanned by species present; and higher Shannon index scores, a measure of species count and abundance (**Figure 2E**; p = 6.45e-10) (Shannon, 1948).

Alpha diversity did not differ significantly between ticks infected versus uninfected with *B. burgdorferi* based on either absolute number of species (**Figure 2B**; p = 0.301) or Faith phylogenetic diversity index score (**Figure 2D**; p = 0.56). *B.burgdorferi*-infected ticks had a slightly higher Shannon index score (**Figure 2F**; p = 0.024), but this difference disappeared after removing *B. burgdorferi* reads (p = 0.58).

**Figure 3:**
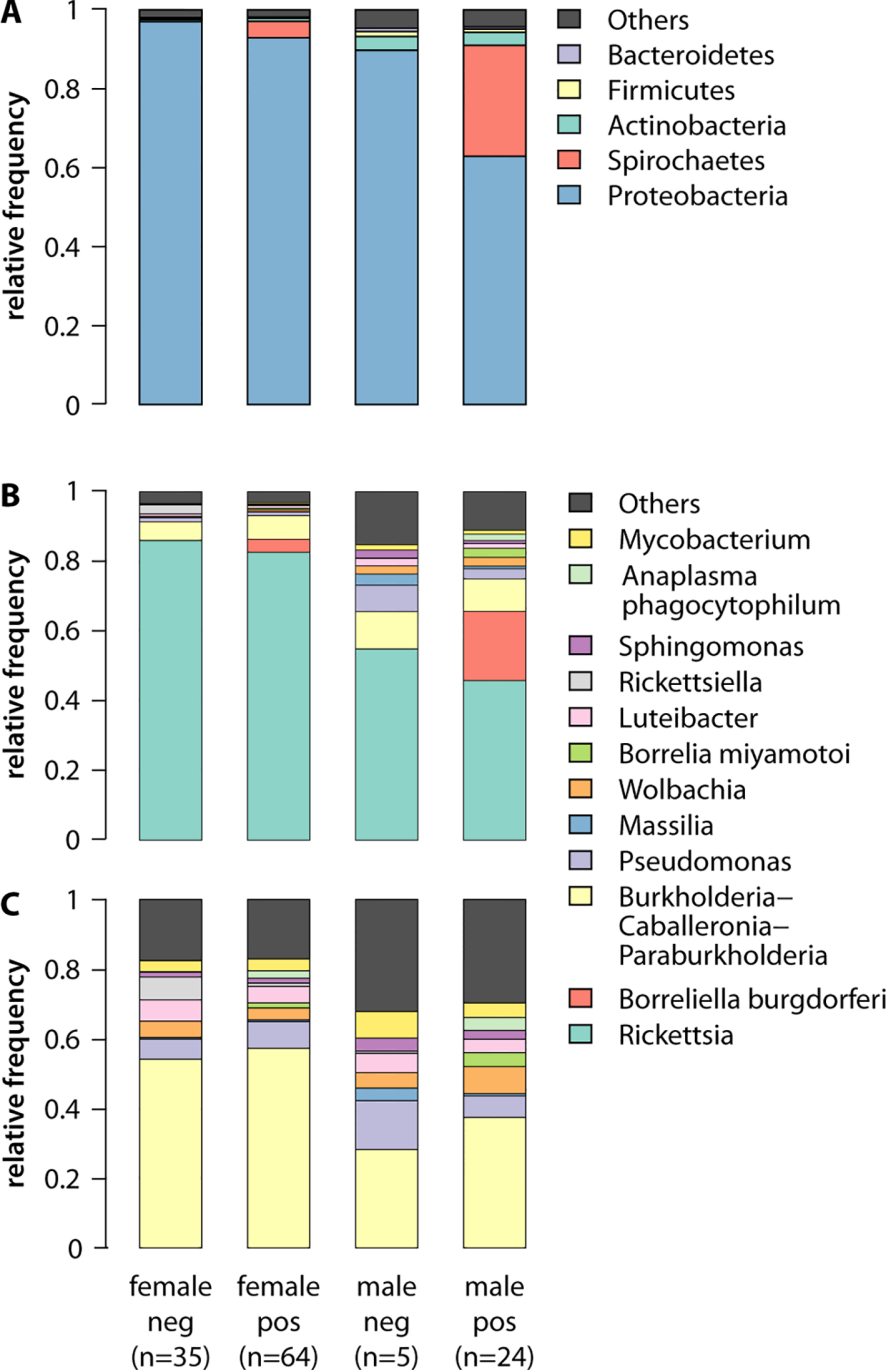
Abundance of microbial taxa in male and female ticks with and without *B. burgdorferi* infection reflects the greater diversity of the male microbiome. **(A)** At the phylum level, *B. burgdorferi* is evident in the abundance of Spirochaetes in the infected ticks, and males have higher frequency non-Proteobacteria phyla, reflecting the greater diversity of their microbiome. Here, “Others” (grey) represents all reads unclassifiable using the 16S SILVA reference database with a 99% identity cutoff. **(B)** The greater microbiome diversity in males, and higher abundance *B. burgdorferi* in infected males (19.89% vs 3.75%; t-test = 4.0505, p = 0.00048), is also evident at the level of genera. Females have a higher frequency of *Rickettsia*, potentially due to the high abundance of that genus in the ovaries(Noda et al., 1997). **(C)** The greater diversity of male microbiome on the genera level persists even when both *B. burgdorferi* and *Rickettsia* are removed from the analysis.

The greater alpha diversity in male ticks was evident in the phylum abundance of the microbiomes (**Figure 3A**). Proteobacteria comprised the highest relative abundance in all ticks, averaging 89.7 in *B. burgdorferi-*uninfected male ticks, 62.9 in infected male ticks, 97.07 in uninfected female ticks, and 92.9% in infected female ticks. The differences between *B. burgdorferi* infected and uninfected *I. scapularis* ticks of both sexes, however, can be explained by the different abundance of Spirochaetes, the phylum that includes *B. burgdorferi*. Among infected ticks, Spirochaetes was the second most abundant phylum, averaging 28.1 in males and 4.22% in females; however, this phylum was essentially absent in uninfected ticks (0.062 in males and 0.034% in females). Genera-level analysis confirmed that infected male ticks had a significantly higher abundance of *B. burgdorferi* than that in infected females (19.89 vs 3.75%; Welch's t-test = 4.0505, p = 0.00048), and that most of the Spirochaetes in the infected ticks were *B. burgdorferi* (**Figure 3B**). After removing *B. burgdorferi* from the analysis, infected and uninfected ticks had similar microbiome profiles on the genera-level, with a much higher proportion of *Rickettsia* in female ticks, consistent with other published results (Van Treuren et al., 2015; Thapa et al., 2018). However, *Rickettsia* did not explain all of the differences between males and female ticks. Even after removing *B. burgdorferi* and Rickettsia species, the diversity in the male ticks remained significantly higher than that in female ticks (**Figure 3C**).

#### Beta diversity

**Figure 4:**
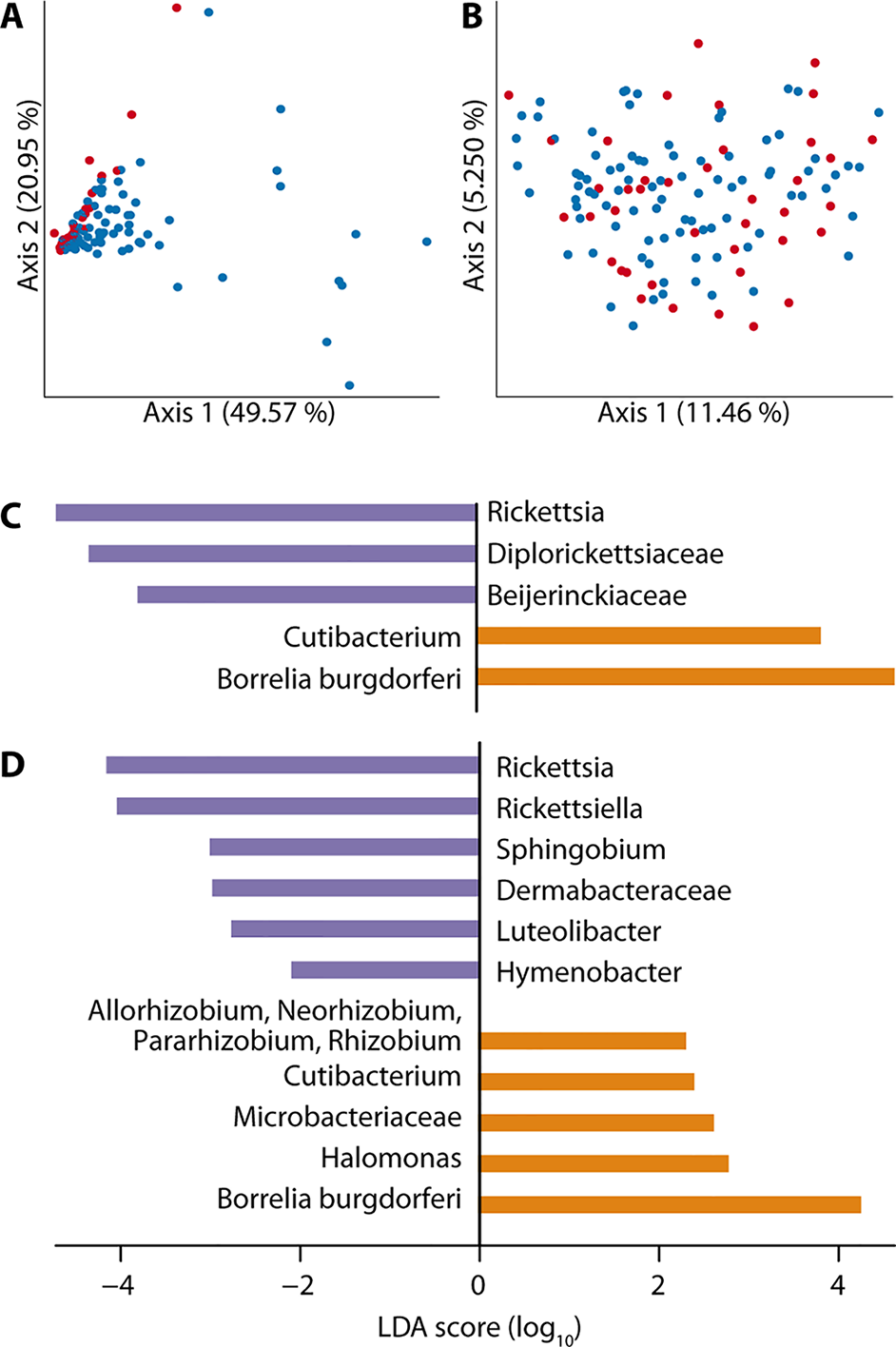
Overall beta diversity of the microbiome does not differ by *B. burgdorferi* infection status; however, specific taxa are significantly over and underrepresented in infected ticks. **(A)** Beta diversity, measured by weighted Unifrac distance, differed significantly between infected (blue) and uninfected (red) ticks (pseudo-F test = 8.28366, p = 0.001), but **(B)** this difference disappears once *B. burgdorferi* is removed from the analysis (pseudo-F test = 1.53959, p = 0.206). **(C)** LEfSe discriminant analysis shows that reads from *B. burgdorferi* and *Cutibacterium* are significantly more abundant (orange) in *B. burgdorferi-*positive ticks, while reads from three taxa are significantly less abundant (purple), including *Rickettsia*. **(D)** The underabundance of *Rickettsia* persists in LEfSe analysis of female ticks only, and the number of significantly more (orange; 5 taxa) and less (purple; 6 taxa) abundant taxa increases.

We also observed differences in the microbiome profiles of *B.burgdorferi*-positive and -negative ticks, with a number of specific taxa over and underrepresented in the infected ticks. Beta diversity differed significantly between infected and uninfected ticks when measured by relative abundance (weighted Unifrac distance (Lozupone et al., 2007), pseudo-F test = 8.28366, p = 0.001; **Figure 4A**). However, there was no difference when beta diversity was measured by occurrence (unweighted UniFrac distance (Lozupone and Knight, 2005), pseudo-F test = 1.197, p = 0.161; **Figure S2**) or when measured as relative abundance after *B. burgdorferi* was removed from the analysis (pseudo-F test = 1.53959, p = 0.206; **Figure 4B**).

Our LEfSe discriminant analysis (Segata et al., 2011) revealed previously unreported associations between *B. burgdorferi* infection and the presence of specific microbial taxa. The abundance of reads from two taxa (*B. burgdorferi* itself and the genus *Cutibacterium*) was significantly higher in *B. burgdorferi-*positive ticks, while reads from three taxa (genus *Rickettsia*, family *Diplorickettsiaceae*, and family *Beijerinckiaceae*) were significantly less abundant (**Figure 4C**). When LEfSe analysis was restricted to female ticks, the number of taxa significantly more or less abundant in *B. burgdorferi-*infected ticks increased markedly (**Figure 4D**). We observed *Rickettsiella* to be more abundant in *B. burgdorferi*-negative female ticks, but this pattern was largely driven by a single uninfected tick with an exceptionally high abundance of *Rickettsiella* (79.4%, compared to 0–3.6% in other ticks). *Rickettsiella* is an intracellular bacterium reported to be pathogenic in some arthropods, but its pathogenicity in ticks is unknown. Finally, we searched for bacterial species known to be pathogenic in humans and detected four pathogens not previously reported to be associated with ticks (**Table 2**).

**Table 2:** Microbiome analysis detected bacterial species pathogenic to humans and not previously reported in ticks.

Pathogen	# of ticks infected	# coinfected with *B. burgdorferi*	Description
*Chryseobacterium indologenes*	3 (2.3%)	2	Opportunistic; can infect immunocompetent humans; exhibits resistance to multiple antibiotics (Hsueh et al., 1996; Cascio et al., 2005; Lin et al., 2010; Chou et al., 2011; McKew, 2014)
*Pseudomonas putida*	22 (17.2%)	14	Associated with trauma or immunocompromised state; lethal in rare cases (Carpenter et al., 2008; Thomas et al., 2013)
*Rhodococcus erythropolis*	15 (11.7%)	10	Infects immunocompromised individuals (Vernazza et al., 1991; Bagdure et al., 2012)
*Streptococcus suis*	2 (1.6%)	2	Can cause systemic disease in humans (Huong et al., 2014)

## Discussion

Efforts to control the transmission and spread of TBD have typically been limited by the lack of data on the microbiome and on the presence, abundance, and dynamics of pathogens in individual ticks. We sought to develop a robust platform, Project Acari, to provide large-scale, high-resolution data on TBP. We focused initially on four essential components: (1) enrollment of large numbers of volunteers through social media; (2) economical collection of ticks that can provide high-quality DNA, regardless of distance from research site; (3) laboratory methods for tick processing that are readily scalable to thousands of samples; and (4) a pilot genomic dataset to validate essential findings of earlier studies and offer new insights.

The results of the first phase of Project Acari underscore the power of citizen science as a tool for sampling large numbers of ticks from geographically diverse locations. Using our custom-designed Tick Kit, we collected nearly 2,500 ticks from across the United States, including individuals of diverse species at various life stages (**Figure 1**). Through our pilot project, we processed 192 *I. scapularis* ticks collected by volunteers in and around Grafton, Massachusetts. This area is reported to have rates of *B. burgdorferi* infection in the *I. scapularis* tick population exceeding 50%, suggesting that we would have sufficiently high infection rates in our pilot sample set for comparison of microbiomes between infected and uninfected ticks (Laboratory of Medical Zoology, U Mass Amherst, 2013). We validated previously reported correlations between tick sex and microbiome profiles, discovered significant associations between specific microbiome components and *B. burgdorferi* infection, and detected human pathogens that had not previously been reported to be carried by ticks.

As part of this pilot project, we developed methods for higher-throughput processing of ticks. One major challenge was simply penetrating the small, hard tick exoskeleton without damaging the DNA. Previous studies used microdissection, bead beating, or both, to homogenize ticks (Moreno et al., 2006; Crowder et al., 2010; Budachetri et al., 2014; Narasimhan et al., 2014; Ammazzalorso et al., 2015). While these approaches are effective, they are also labor intensive, imposing severe constraints on the total number of individual ticks that can be processed. Bead beating, for example, requires the purchase of a specialized instrument that can achieve speeds of at least 3,500 rpm. Moreover, the commercially available machines able to operate at such a high speed have maximum capacity of 24 individual ticks. Thus, while effective for tick homogenization, bead-beating is not readily scalable for processing large numbers of ticks in parallel.

Our new method is simple. We first place a single tick in each well of a 96-well deep well plate, then flash-freeze the ticks by placing the plate in liquid nitrogen. We then use the Zymo Research Squisher-96 to shatter and homogenize the ticks. Our protocol builds on earlier work using liquid nitrogen (Hill and Gutierrez, 2003; Steiner et al., 2008) and is consistent, scalable, and low-cost. The 96-well plate format integrates easily into high-throughput pipelines, and, by freezing multiple 96-well plates in a single large container, we can simultaneously homogenize hundreds of ticks in under 10 min, providing the rapid throughput needed for large-scale tick genomics. We confirmed that this method could be used to extract high-quality DNA both from the 192 ticks in the pilot set and from 188 ticks provided by volunteers who mailed back our custom-designed Tick Card.

In our pilot genomics project, we determined *B.burgdorferi* infection status and 16S microbiome sequence data for 128 wild-caught *I. scapularis* ticks. We validated previously published findings of overall higher microbial diversity in males than in females and the higher abundance of *Rickettsia* in the female ticks in both laboratory and wild-caught samples (Van Treuren et al., 2015; Zolnik et al., 2016; Thapa et al., 2018). The abundance of *Rickettsia* in the ovaries of female ticks may play an important developmental role (Noda et al., 1997). In contrast to previous findings in laboratory-raised ticks, we found that the differences in the microbiomes of our male and female wild-caught ticks persisted even after removing *Rickettsia* reads (**Figure 3C**) (Thapa et al., 2018).

Studying wild-caught ticks provides a real-world context for findings made using laboratory-reared ticks. For example, previous work showed that *Borrelia* did not colonize ticks raised in sterile environments and with very low microbiome diversity (Narasimhan et al., 2014). In our field-collected samples, however, we observed no correlation between microbiome diversity and *Borrelia* infection status, suggesting that wild-caught ticks have sufficient diversity to allow *Borrelia* colonization. Thus, while lab-reared ticks can provide insight into the dynamics of TBPs not accessible using field-collected samples, TBP interventions developed based on findings from laboratory ticks may not be readily applicable to real-world populations.

With microbiome data from just 128 ticks, we discovered components correlated with infection status that had not been previously described, highlighting the need for larger-scale tick research. Our findings also illustrate why, even as projects scale up, data at single-tick resolution remain essential. Our finding of significantly higher abundance of *Ricketsiella* in uninfected female ticks was driven almost entirely by a single tick with an exceptionally high abundance of *Rickettsiella* (79.4%, compared to 0–3.6% in other ticks), a phenomenon only detectable because we were able to distinguish the DNA from individual ticks. The capacity of our approach to process a large number of ticks and to sample from many different geographic locations also revealed the presence of some human pathogens not previously associated with ticks; however, further work will be needed to determine the frequencies of these associations.

Data at single-tick resolution also enabled us to detect 13 instances of ticks infected with multiple pathogens. Co-infection is well-documented in TBPs, with up to 40% of ticks reported to carry multiple pathogens (Stromdahl et al., 2001; Swanson et al., 2006; Steiner et al., 2008; Hersh et al., 2014; Diuk-Wasser et al., 2016; Nieto et al., 2018). The transmission of multiple pathogens can increase both the duration and intensity of TBD symptoms and complicate diagnosis (Sanchez et al., 2016). Understanding the prevalence of TBP co-infection in the tick population for a given region could immediately impact diagnostic approaches and treatment decisions.

We also detected bacterial species known to be pathogenic in humans, including four not previously reported to be associated with ticks (**Table 2**). Three of these, *Chryseobacterium indologenes (* Hsueh et al., 1996 *)*, *Pseudomonas putida* (Thomas et al., 2013), and *Rhodococcus erythropolis (* de Carvalho and da Fonseca, 2005 *)*, are environmental microbes and opportunistic human pathogens. They may have colonized the ticks following environmental exposure (Narasimhan et al., 2014) or upon contact with vertebrate hosts (Narasimhan and Fikrig, 2015; Bonnet et al., 2017). The fourth of these microbes, *Streptococcus suis*, is a pig pathogen and cause of zoonotic disease (Lun et al., 2007; Dejace et al., 2017). This pathogen is found in the northeastern United States, where it infects and sickens swine workers, and the tick could have acquired it either through feeding on an infected animal or through environmental exposure (Smith et al., 2008; Soares et al., 2015; Dejace et al., 2017).

The methods we present here enable large-scale, high-throughput tick genomics, but have several limitations. First, homogenizing whole ticks provides no information on associations between specific microbes and specific tick organs. Microdissection will be required to investigate the possibility that microbes in sex-specific organs underlie the greater microbial diversity we observed in male ticks (Narasimhan et al., 2014). In addition, while we requested volunteers send only ticks found unattached, and excluded on receipt any ticks visibly blood engorged, we cannot completely exclude the possibility of an earlier blood meal influencing the microbial profile of a given tick (Rynkiewicz et al., 2015). The next phase of Project Acari will address this limitation by incorporating assays to detect vertebrate DNA indicative of prior feeding (Reeves et al., 2018). Finally, our citizen science-based design, while allowing the collection of very large numbers of ticks, does not ensure uniform collection procedures and the geographic distribution of sampling is sensitive to variation in volunteer engagement. Limitations in what citizen scientists can collect can preclude studies of TBD dynamics (detailed surveys of specific areas, vertical transmission and host-pathogen concordance) which may be best addressed using smaller cohorts of ticks bred in the lab or wild-caught by experienced researchers (Clay et al., 2008; Rynkiewicz et al., 2015).

The next phase of Project Acari will screen ticks for a broader set of pathogens and use genomics to collect detailed information on the sex, species, and genomic variants of each tick. The work reported here assessed tick species and sex through visual inspection. This approach is laborious and potentially error-prone and would ideally be replaced by genomic approaches. Relative to the intense focus on using genomics for controlling diseases transmitted by other arthropod vectors (Hill et al., 2005), tick genomics is much less developed. Only a single species, *I. scapularis*, has a reference genome, and sequences are not yet anchored to chromosomes (Gulia-Nuss et al., 2016). Without high quality genome assemblies for tick vector species, we cannot easily identify genomic markers that indicate tick species and sex, or capture known pesticide-resistance loci. Thus, there is an urgent need for “genomic infrastructure” to enable the development of new technologies for TBP surveillance and interventions.

TBPs are increasing in prevalence worldwide, likely exacerbated by climate change (USGCRP, 2016). Here, we have demonstrated the potential of citizen science to support large-scale, longitudinal TBP genomic analysis across broad geographic areas. Through Project Acari, we collected ticks from across the United States and also resampled the same locations in multiple years. While the initial phase described here focused on a single tick species and pathogen, our genomic approaches are readily extendable to other tick and pathogen species. We envision Project Acari as an open data resource that engages directly with communities at risk from TBP to provide real-time information on the spread of pathogens, provide a rich source of data for investigating the factors that drive this spread, and inform the development of new control strategies.

## Data Availability Statement

The 16S sequencing data generated for the tick samples described in **Table S1** can be found in the NCBI Sequence Read Archive under NCBI BioProject: PRJNA527832: Ixodes scapularis 16S microbiome survey.

## Ethics Statement

This study did not require approval by an Institutional Review Board as is does not involve Human Subjects as defined by the U.S. Department of Health & Human Service Office for Human Research Protections. No data about living individuals or identifiable private information was used in the research.

## Author Contributions

GC, JB, DG, and EK designed the study. GC, JM, JH, and EK developed the citizen science approach. GC and PM designed and implemented laboratory methods. GC performed computational analysis and statistical analyses with guidance from JB. GC, RD, DG, JB, and EK wrote the article.

## Funding

The project was supported by a Bioinformatics and Integrative Biology Program Catalytic Research Grant and Dr. Karlsson's start-up funding from the University of Massachusetts Medical School.

## Conflict of Interest

The authors declare that the research was conducted in the absence of any commercial or financial relationships that could be construed as a potential conflict of interest.
