# Supplementary material for: Combining Citizen Science and Genomics to Investigate Tick, Pathogen, and Commensal Microbiome at Single-Tick Resolution
Source: Front Genet. 2020 Jan 21;10:1322. doi: 10.3389/fgene.2019.01322 (PMC6985576; doi:10.3389/fgene.2019.01322)
Supplement: Supplementary file 1 [file DataSheet_1.pdf]

Table S1. 192 Ticks included in pilot study, with microbiome DNA and library yield.

N	Sample ID	Borrelia (PCR)	Gender	DNA conc (ng/ul)	DNA yield (ng)	Lib yield (nM)	16S sequencing?
1	1A1	Neg	F	18.36	734.4	43.5	yes
2	1A2	Neg	F	15.42	616.8	33.9	yes
3	1A3	Pos	F	47.19	1887.6	55.8	yes
4	1A4	Neg	F	28.44	1137.6	82.0	yes
5	1A5	Pos	F	7.79	311.6	16.1	yes
6	1A6	Pos	F	30.4	1216	49.1	yes
7	1A7	Neg	F	40.72	1628.8	34.4	yes
8	1A8	Pos	F	25.3	1012	25.7	yes
9	1A9	Pos	F	73.99	2959.6	10.3	yes
10	1A10	Pos	F	22.93	917.2	42.8	yes
11	1A11	Pos	F	29.67	1186.8	30.1	yes
12	1A12	Pos	F	13.54	541.6	70.0	yes
13	1B1	Neg	F	20.46	818.4	29.1	yes
14	1B2	Neg	F	13.57	542.8	12.5	yes
15	1B3	Pos	F	18.77	750.8	20.0	yes
16	1B4	Neg	F	6.34	253.6	45.5	yes
17	1B5	Neg	F	18.05	722	22.6	yes
18	1B6	Neg	F	28.04	1121.6	18.3	yes
19	1B7	Neg	F	22.96	918.4	16.8	yes
20	1B8	Pos	F	22.57	902.8	16.6	yes
21	1B9	Pos	F	17.89	715.6	38.7	yes
22	1B10	Pos	F	24.65	986	26.5	yes
23	1B11	Neg	F	29.26	1170.4	29.1	yes
24	1B12	Neg	F	24.98	999.2	36.8	yes
25	1C1	Pos	F	50.65	2026	25.7	yes
26	1C2	Neg	F	37.55	1502	29.8	yes
27	1C3	Neg	F	42.07	1682.8	32.2	yes
28	1C4	Neg	F	25.92	1036.8	51.0	yes
29	1C5	Pos	F	58.97	2358.8	13.9	yes
30	1C6	Neg	F	28.71	1148.4	26.5	yes
31	1C7	Neg	F	14.16	566.4	35.8	yes
32	1C8	Pos	F	22.72	908.8	7.7	no
33	1C9	Pos	F	19.82	792.8	32.0	yes
34	1C10	Pos	F	8.26	330.4	9.4	no
35	1C11	Neg	F	15.18	607.2	29.8	yes
36	1C12	Neg	F	42.49	1699.6	27.2	yes
37	1D1	Pos	F	37.85	1514	38.7	yes
38	1D2	Pos	F	24.9	996	13.2	yes
39	1D3	Neg	F	21.27	850.8	58.2	yes
40	1D4	Neg	F	16.62	664.8	19.2	yes

Table S1. 192 Ticks included in pilot study, with microbiome DNA and library yield.

N	Sample ID	Borrelia (PCR)	Gender	DNA conc (ng/ul)	DNA yield (ng)	Lib yield (nM)	16S sequencing?
41	1D5	Pos	F	41.53	1661.2	6.0	no
42	1D6	Pos	F	34.03	1361.2	8.2	no
43	1D7	Neg	F	4.38	175.2	7.5	no
44	1D8	Neg	F	15.27	610.8	6.0	no
45	1D9	Neg	F	22.01	880.4	26.5	yes
46	1D10	Pos	F	76.24	3049.6	6.0	no
47	1D11	Pos	F	15.35	614	31.0	yes
48	1D12	Pos	F	32.71	1308.4	30.3	yes
49	1E1	Neg	F	48.43	1937.2	31.7	yes
50	1E2	Pos	F	21.37	854.8	39.2	yes
51	1E3	Pos	F	39.2	1568	31.5	yes
52	1E4	Pos	F	41.9	1676	5.5	no
53	1E5	Pos	F	19.79	791.6	6.3	no
54	1E6	Pos	F	37.82	1512.8	13.0	yes
55	1E7	Neg	F	44.95	1798	8.7	no
56	1E8	Pos	F	33.49	1339.6	11.5	yes
57	1E9	Neg	F	11.47	458.8	24.3	yes
58	1E10	Neg	F	10.62	424.8	25.0	yes
59	1E11	Neg	F	43.35	1734	9.6	no
60	1E12	Pos	F	38.9	1556	36.8	yes
61	1F1	Pos	F	24.65	986	31.0	yes
62	1F2	Neg	F	39.03	1561.2	27.2	yes
63	1F3	Pos	F	62.02	2480.8	30.3	yes
64	1F4	Pos	F	10.03	401.2	22.6	yes
65	1F5	Pos	F	29.82	1192.8	11.3	yes
66	1F6	Pos	F	33.36	1334.4	11.5	yes
67	1F7	Pos	F	17.73	709.2	14.2	yes
68	1F8	Neg	F	9.67	386.8	5.3	no
69	1F9	Neg	F	37.42	1496.8	10.3	yes
70	1F10	Neg	F	11.14	445.6	8.4	no
71	1F11	Pos	F	27.07	1082.8	24.5	yes
72	1F12	Pos	F	20.26	810.4	36.6	yes
73	1G1	Neg	F	22.4	896	28.1	yes
74	1G2	Pos	F	23.1	924	18.5	yes
75	1G3	Pos	F	18.52	740.8	35.8	yes
76	1G4	Pos	F	29.61	1184.4	11.5	yes
77	1G5	Pos	F	35.19	1407.6	12.0	yes
78	1G6	Neg	F	44.31	1772.4	7.2	no
79	1G7	Pos	F	55.95	2238	6.0	no
80	1G8	Neg	F	26.9	1076	5.5	no

Table S1. 192 Ticks included in pilot study, with microbiome DNA and library yield.

N	Sample ID	Borrelia (PCR)	Gender	DNA conc (ng/ul)	DNA yield (ng)	Lib yield (nM)	16S sequencing?
81	1G9	Pos	F	10.53	421.2	27.9	yes
82	1G10	Neg	F	26.18	1047.2	3.4	no
83	1G11	Neg	F	19.25	770	29.8	yes
84	1G12	Pos	F	13.92	556.8	53.6	yes
85	1H1	Pos	F	26.08	1043.2	39.4	yes
86	1H2	Pos	F	14.03	561.2	40.9	yes
87	1H3	Neg	F	22.48	899.2	52.9	yes
88	1H4	Pos	F	12.18	487.2	17.8	yes
89	1H5	Pos	F	6.08	243.2	41.6	yes
90	1H6	Neg	F	19.44	777.6	41.8	yes
91	1H7	Pos	F	19.81	792.4	24.8	yes
92	1H8	Pos	F	11.6	464	28.9	yes
93	1H9	Pos	F	17.74	709.6	25.7	yes
94	1H10	Neg	F	53.13	2125.2	7.2	no
95	1H11	Pos	F	17.2	688	31.0	yes
96	1H12	Pos	F	47.19	1887.6	49.1	yes
97	2A1	Pos	F	19.29	771.6	18.5	yes
98	2A2	Pos	F	16.34	653.6	24.1	yes
99	2A3	Pos	F	18.54	741.6	45.2	yes
100	2A4	Pos	M	8.44	337.6	16.1	yes
101	2A5	Neg	M	5.06	202.4	2.4	no
102	2A6	Pos	M	11.01	440.4	12.3	yes
103	2A7	Pos	M	8.67	346.8	11.1	yes
104	2A8	Pos	M	9.05	362	11.8	yes
105	2A9	Neg	M	7.17	286.8	15.2	yes
106	2A10	Pos	M	8.24	329.6	19.2	yes
107	2A11	Pos	M	13.58	543.2	21.2	yes
108	2A12	Pos	M	7.18	287.2	20.9	yes
109	2B1	Pos	F	19.21	768.4	28.1	yes
110	2B2	Pos	F	19.83	793.2	18.8	yes
111	2B3	Neg	F	15.04	601.6	25.0	yes
112	2B4	Pos	M	9.4	376	8.7	no
113	2B5	Pos	M	7.22	288.8	5.3	no
114	2B6	Neg	M	23.97	958.8	1.2	no
115	2B7	Pos	M	7.23	289.2	7.7	no
116	2B8	Pos	M	12.26	490.4	10.6	yes
117	2B9	Pos	M	11.57	462.8	16.6	yes
118	2B10	Pos	M	13.96	558.4	6.0	no
119	2B11	Pos	M	11.95	478	12.0	yes
120	2B12	Pos	M	13.32	532.8	15.6	yes

Table S1. 192 Ticks included in pilot study, with microbiome DNA and library yield.

N	Sample ID	Borrelia (PCR)	Gender	DNA conc (ng/ul)	DNA yield (ng)	Lib yield (nM)	16S sequencing?
121	2C1	Pos	F	5.04	201.6	18.8	yes
122	2C2	Pos	F	17.97	718.8	15.2	yes
123	2C3	Pos	F	17.29	691.6	44.0	yes
124	2C4	Pos	M	10.17	406.8	7.0	no
125	2C5	Neg	M	17.31	692.4	8.4	no
126	2C6	Pos	M	9.02	360.8	4.3	no
127	2C7	Neg	M	20.72	828.8	1.7	no
128	2C8	Pos	M	16.82	672.8	2.9	no
129	2C9	Neg	M	9.86	394.4	11.8	yes
130	2C10	Neg	M	10.36	414.4	6.5	no
131	2C11	Neg	M	5.61	224.4	18.3	yes
132	2C12	Neg	M	17.16	686.4	10.1	yes
133	2D1	Pos	F	18.94	757.6	28.4	yes
134	2D2	Neg	F	15.76	630.4	10.3	yes
135	2D3	Pos	F	32.21	1288.4	15.4	yes
136	2D4	Pos	M	11.5	460	5.5	no
137	2D5	Neg	M	21.66	866.4	4.6	no
138	2D6	Neg	M	22.7	908	4.8	no
139	2D7	Pos	M	10.49	419.6	3.1	no
140	2D8	Neg	M	5.62	224.8	1.2	no
141	2D9	Pos	M	13.82	552.8	5.1	no
142	2D10	Pos	M	15.96	638.4	12.7	yes
143	2D11	Pos	M	10.49	419.6	10.6	yes
144	2D12	Pos	M	20.74	829.6	17.8	yes
145	2E1	Neg	F	39.48	1579.2	36.8	yes
146	2E2	Pos	F	23.14	925.6	15.9	yes
147	2E3	Neg	F	11.69	467.6	20.9	yes
148	2E4	Pos	M	13.81	552.4	8.7	no
149	2E5	Neg	M	17.58	703.2	5.5	no
150	2E6	Pos	M	24.61	984.4	5.3	no
151	2E7	Neg	M	3.88	155.2	1.9	no
152	2E8	Neg	M	2.87	114.8	2.4	no
153	2E9	Pos	M	11.94	477.6	14.4	yes
154	2E10	Pos	M	12.58	503.2	8.2	no
155	2E11	Neg	M	19.51	780.4	7.9	no
156	2E12	Pos	M	27.74	1109.6	28.6	yes
157	2F1	Pos	F	26.15	1046	14.7	yes
158	2F2	Pos	F	20.7	828	18.8	yes
159	2F3	Pos	M	9.18	367.2	8.4	no
160	2F4	Pos	M	4.61	184.4	6.3	no

Table S1. 192 Ticks included in pilot study, with microbiome DNA and library yield.

N	Sample ID	Borrelia (PCR)	Gender	DNA conc (ng/ul)	DNA yield (ng)	Lib yield (nM)	16S sequencing?
161	2F5	Neg	M	1.86	74.4	2.4	no
162	2F6	Neg	M	21.37	854.8	2.6	no
163	2F7	Pos	M	16.89	675.6	2.6	no
164	2F8	Neg	M	5.26	210.4	10.1	yes
165	2F9	Neg	M	7.39	295.6	9.9	no
166	2F10	Pos	M	19.73	789.2	4.1	no
167	2F11	Pos	M	18.94	757.6	8.2	no
168	2F12	Pos	M	14.12	564.8	13.9	yes
169	2G1	Pos	F	23.34	933.6	33.7	yes
170	2G2	Pos	F	8.46	338.4	37.8	yes
171	2G3	Pos	M	14.14	565.6	16.1	yes
172	2G4	Pos	M	14.68	587.2	11.1	yes
173	2G5	Neg	M	16.32	652.8	6.7	no
174	2G6	Neg	M	14.33	573.2	9.4	no
175	2G7	Neg	M	2.37	94.8	3.6	no
176	2G8	Neg	M	3.32	132.8	2.9	no
177	2G9	Pos	M	18.73	749.2	8.2	no
178	2G10	Pos	M	19.13	765.2	10.3	yes
179	2G11	Pos	M	14.41	576.4	12.3	yes
180	2G12	Neg	M	2.59	103.6	8.7	no
181	2H1	Pos	F	16.92	676.8	38.0	yes
182	2H2	Pos	F	22.08	883.2	26.0	yes
183	2H3	Pos	M	13.73	549.2	22.4	yes
184	2H4	Neg	M	12.63	505.2	9.1	no
185	2H5	Pos	M	15.56	622.4	12.0	yes
186	2H6	Pos	M	23.49	939.6	5.3	no
187	2H7	Neg	M	13.24	529.6	1.2	no
188	2H8	Pos	M	2.8	112	1.2	no
189	2H9	Pos	M	59.95	2398	3.8	no
190	2H10	Neg	M	14.29	571.6	6.3	no
191	2H11	Pos	M	14.65	586	13.7	yes
192	2H12	Pos	M	19.66	786.4	7.0	no
