# Supplementary material for: Combining Citizen Science and Genomics to Investigate Tick, Pathogen, and Commensal Microbiome at Single-Tick Resolution
Source: Front Genet. 2020 Jan 21;10:1322. doi: 10.3389/fgene.2019.01322 (PMC6985576; doi:10.3389/fgene.2019.01322)

Figure S1. Mean number of ASVs observed for male and female ticks across a range of sequencing depths. For both males and females, rarefaction curves at depth from 1 to 20,000 sequences show no significant increase in the number of ASVs observed at sequencing depths beyond 8000 reads/sample (in total, 4,176,619 sequences and 1,420 unique ASVs).

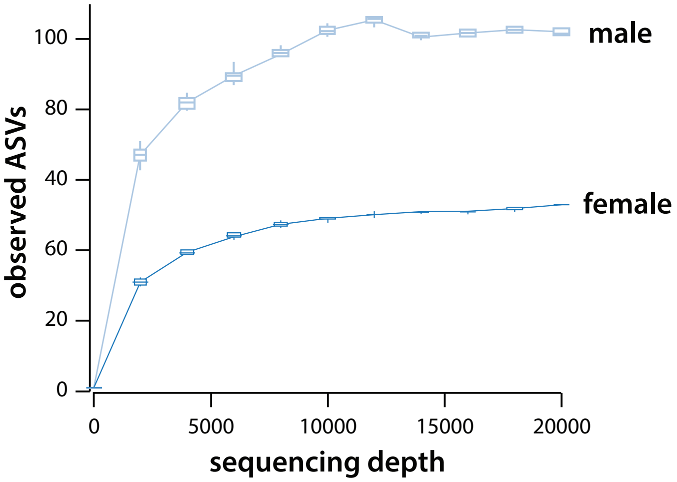

Supplement: Supplementary file 2 [file Image_1.pdf]
