# Supplementary material for: Combining Citizen Science and Genomics to Investigate Tick, Pathogen, and Commensal Microbiome at Single-Tick Resolution
Source: Front Genet. 2020 Jan 21;10:1322. doi: 10.3389/fgene.2019.01322 (PMC6985576; doi:10.3389/fgene.2019.01322)

Figure S2. Beta diversity measured by occurence. Beta diversity did not differ significantly between B. burgdorferi-infected (blue) and uninfected (red) ticks when measured by occurrence (unweighted UniFrac distance, pseudo-F test=1.197, p=0.161).

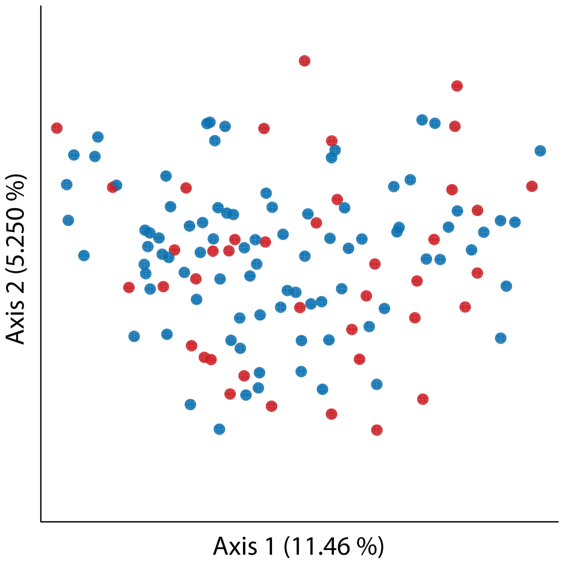

Supplement: Supplementary file 3 [file Image_2.pdf]
